# Preoperative CT-Guided Near-Infrared Dye Marking for Thoracoscopic Resection of Pulmonary Nodules: A Case Report

**DOI:** 10.3389/fsurg.2022.919227

**Published:** 2022-06-08

**Authors:** Emanuele Voulaz, Alberto Testori, Felice D'Antuono, Umberto Cariboni, Marco Alloisio, Giuseppe Mangiameli

**Affiliations:** ^1^ Department of Biomedical Sciences, Humanitas University, Rozzano, Italy; ^2^ Division of Thoracic Surgery, IRCCS Humanitas Research Hospital, Rozzano, Italy; ^3^ Divisioni of Diagnostic and Interventional Radiology, IRCCS Humanitas Research Hospital, Rozzano, Italy

**Keywords:** preoperative marking for pulmonary nodule, indocyanin green (ICG), lung resection, lung metastases, CT guide

## Abstract

Localization of small-sized pulmonary nodules is challenging during video-assisted thoracoscopic surgery. Several preoperative strategies have been developed to mark these targets. We describe our localization strategy using a preoperative computed tomography-guided near-infrared dye marking.

## Introduction

Minimally invasive surgery, including video-assisted thoracoscopic surgery (VATS), has been ideal for the resection of small nodules because it results in minimal postoperative morbidity and mortality, less pain, and a better quality of life than with an open thoracotomy (1, 2).

Localizing small-sized pulmonary nodules is challenging during VATS. Thus, several preoperative strategies have been developed to mark these targets (3, 4). Inadequate nodule localization may lead to prolonged operative time with an increased risk of performing an unplanned thoracotomy to find the nodule (5–7).

## Case Description

**Figure 1:**
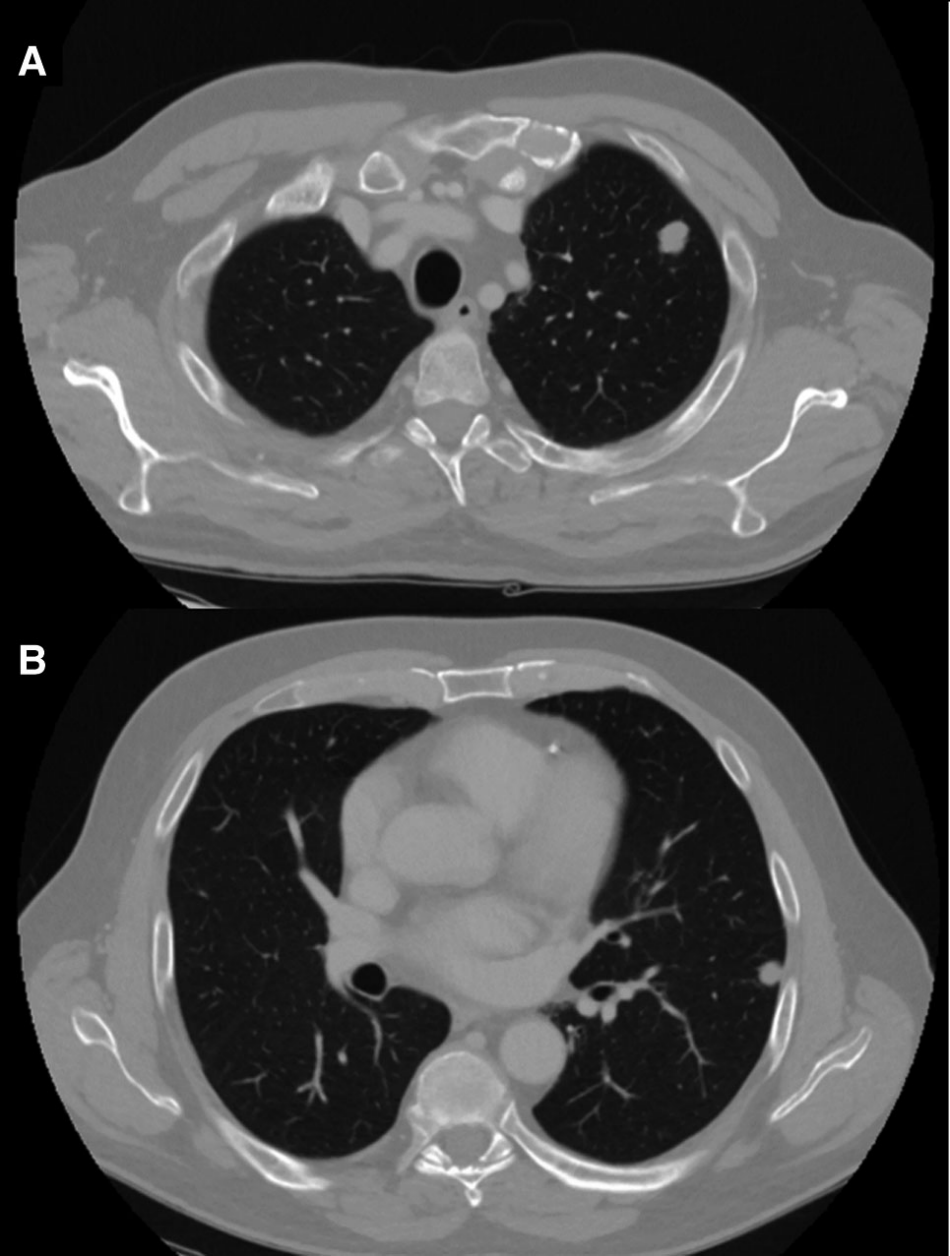
CT scan of two left lung nodules: (**A**) 15 mm upper lobe; and (**B**) 12 mm lower lobe.

We present a case of a no smoker 62-year-old man diagnosed with two metachronous left lung metastases (15 and 12 mm) 20 months after surgery for colorectal adenocarcinoma (Figure 1). After discussion in a multidisciplinary meeting, the patient was scheduled to undergo double video-assisted thoracoscopic wedge resection.

We describe and discuss the technical steps of our localization strategy of nodules localization by using a preoperative CT-guided near-infrared dye marking (NIDM).

**Figure 2:**
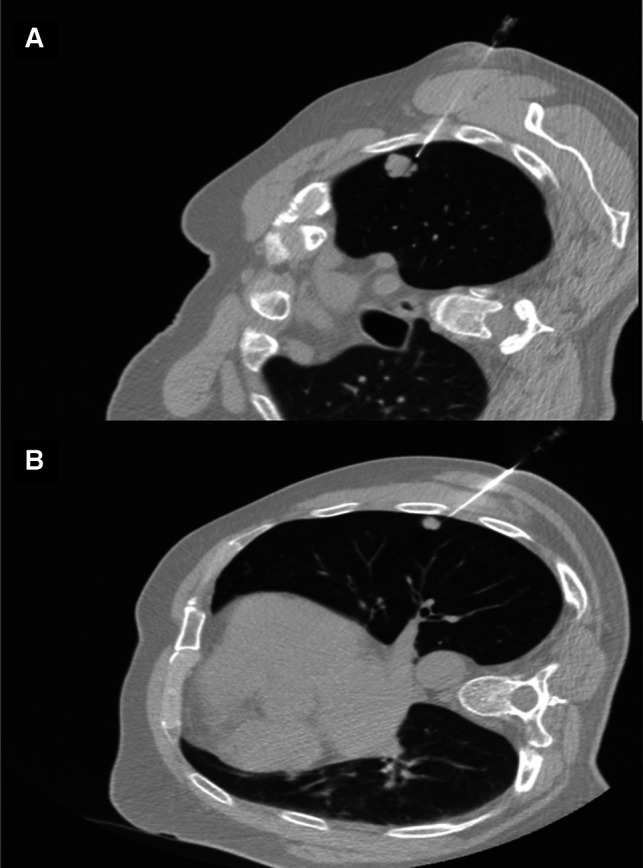
CT scan of needle injection of indocyanine green.

**Figure 3:**
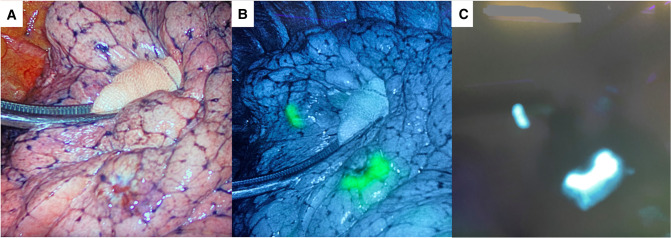
(**A**) Intraoperative field. Metastatic nodules: infrared (**B**) and white light (**C**) signal images.

Written informed consent was obtained from the patient. The first step of the surgery consists of a preoperative CT-guided injection of indocyanine green (ICG). ICG is powder at 50 mg/10 ml and diluted in 20 ml of human albumin 20% (2.5 mg/ml). A 1 ml syringe was filled with the ICG/albumin mixture and connected to a CT-guided percutaneous 21-gauge needle, and the needle lumen was filled with the marking solution prior to the marking procedure. After local anesthesia with lidocaine, a marking solution (1 ml) was injected near the nodules (Figure 2). CT scan was repeated 30 min after the injection to monitor for pneumothorax or air embolism. The entire procedure lasted 60 min and was performed by expert interventional radiologists. Surgery was performed 5 h later. During bi-portal VATS, ICG-FL was visualized using Stryker’s SPY Fluorescence technology with a 30° camera, and the thoracoscopic NIDM detection was immediate (Figure 3). Thoracoscopic instruments were used to clamp the lung to set an imaginary staple line for the intended resection (green area), and wedges resections were performed using manual staplers. After resections, the specimen was observed, and the surgical margin was inspected macroscopically; the operative time was 52 min. The postoperative course was uneventful, and the patient was discharged on day 2. Final pathology confirmed R0 resection for both metastatic nodules.

## Comments

Detection of small pulmonary nodules has increased with the widespread use of chest CT screening (8, 9). Similarly, surgical resection of pulmonary metastases is nowadays widely performed in selected patients (10).

VATS is the minimally invasive procedure of choice in cases involving unsuccessful diagnosis through percutaneous transthoracic needle biopsy or the need for the resection of pulmonary nodules. However, two significant limits are strictly linked to the VATS procedure: the inability to visualize or palpate the target lesions. Furthermore, ground-glass nodule lesions do not alter the surface of the visceral pleura, and the elevation of tumors cannot be perceived in the deflated lung during VATS (11).

To overcome these problems, several localization methods have been developed to mark small-sized nodules before VATS (5–7). The most common markers are hook–thread, spiral wire needle, microcoil, fiducial marker, or dyes (methylene blue); each is injected into the lung near the target using a CT-guided percutaneous injection approach. Alternatively, barium and lipiodol or gamma-emitting radioisotopes can be injected, and the labeled nodules intraoperatively are detected using fluoroscopy or a gamma probe (12). Each of these methods is currently in clinical use but has unique drawbacks.

Kleedehn et al. reported that hook–wire needle localization has major complications like pneumothorax and pulmonary hemorrhage. The consequent dislodgement of the wire may make the surgical resection even more complex (13). The injection of methylene blue provides an effective, safe, and inexpensive method for intrathoracic localization, but diffusion away from the nodule represents the major limitation of this technique (3). Furthermore, radiation exposure is a problem for all radioisotopes. Asamura et al. first in 1994, and Lizza et al. later described a technique that involves the computed tomography-guided coil injection of a metallic coil and subsequent thoracoscopic resection under roentgenographic fluoroscopy (14, 15).

Our technique presents several specific advantages. First is the reproducibility and the absence of specific skills for an interventional radiologist who usually performs percutaneous TC lung biopsies. Second, the risk of radiation exposure is not present. Furthermore, target localization is usually not complicated by displacement or diffusion. ICG injection is well tolerated by the patient, which can freely move without the risk of any landmark dislocation. By the injection of other radiotracers like methylene blue or lipiodol is not easily identified the precise resection margin while has been described that dyes rapidly diffuse and do not remain in the target lesion. Finally, NIDM does not need immediate surgery because the marking persists from hours to days (3).

On the other side, NIDM presents some disadvantages. In cases of severe pulmonary emphysema, there is concern that liquid markers do not form a distinct spot but rather diffuse into pulmonary cysts; therefore, ICG may not be ideal in such cases. Moreover, the VATS equipment required to visualize ICG-FL is expensive and is available only in a limited number of institutions. The above-described procedure is the first European experience, having been exclusively described in oriental series (3, 4). The use of albumin as a solution for ICG is the first report in the literature.

## Conclusion

In our experience, ICG diluted human albumin effectively overcame the diffusion and limitations of most common markers, facilitating accurate localization and resection of pulmonary nodules during VATS. Further investigation, including larger series, could confirm our initial report.

## Data Availability

The raw data supporting the conclusions of this article will be made available by the authors, without undue reservation.

## References

[1] Bendixen M Jørgensen OD Kronborg C Andersen C Licht PB. Postoperative pain and quality of life after lobectomy via video-assisted thoracoscopic surgery or anterolateral thoracotomy for early-stage lung cancer: a randomised controlled trial. Lancet Oncol. (2016) 17(6):836–44. 10.1016/S1470-2045(16)00173-X 27160473

[2] Vannucci F Gonzalez-Rivas D. Is VATS lobectomy standard of care for operable non-small cell lung cancer? Lung Cancer. (2016) 100:114–9. 10.1016/j.lungcan.2016.08.004 27597290

[3] Anayama T Hirohashi K Miyazaki R Okada H Kawamoto N Yamamoto M Near-infrared dye marking for thoracoscopic resection of small-sized pulmonary nodules: comparison of percutaneous and bronchoscopic injection techniques. J Cardiothorac Surg. (2018) 13(1):5. 10.1186/s13019-018-0697-6 29329549 PMC5767012

[4] Yang YL Li1 ZZ Huang WC Zhuang J Lin DY Zhong WZ Electromagnetic navigation bronchoscopic localization versus percutaneous CT-guided localization for thoracoscopic resection of small pulmonary nodules. Thorac Cancer. (2021) 12(4):468–74. 10.1111/1759-7714.13775 33398925 PMC7882377

[5] Suzuki K Nagai K Yoshida J Ohmatsu H Takahashi K Nishimura M Video-assisted thoracoscopic surgery for small indeterminate pulmonary nodules: indications for preoperative marking. Chest. (1999) 115(2):563–8. 10.1378/chest.115.2.563 10027460

[6] Saito H Minamiya Y Matsuzaki I Tozawa K Taguchi K Nakagawa T Indication for preoperative localization of small peripheral pulmonary nodules in thoracoscopic surgery. J Thorac Cardiovasc Surg. (2002) 124(6):1198–202. 10.1067/mtc.2002.127331 12447187

[7] Powell TI Jangra D Clifton JC Lara-Guerra H Church N English J Peripheral lung nodules: fluoroscopically guided video-assisted thoracoscopic resection after computed tomography-guided localization using platinum micro coils. Ann Surg. (2004) 240(3):481–8. 10.1097/01.sla.0000137132.01881.57 15319719 PMC1356438

[8] Crucitti P Gallo IF Santoro G Mangiameli G. Lung cancer screening with low dose CT: experience at Campus Bio-Medico of Rome on 1500 patients. Minerva Chir. (2015) 70(6):393–9. 25700151

[9] Infante M Cavuto S Lutman FR Passera E Chiarenza M Chiesa G Long-term follow-up results of the DANTE trial, a randomized study of lung cancer screening with spiral computed tomography. Am J Respir Crit Care Med. (2015) 191(10):1166–75. 10.1164/rccm.201408-1475OC 25760561

[10] Gonzalez M Zellweger M Nardini M Migliore M. Precision surgery in lung metastasectomy. Future Oncol. (2020) 16(16s):7–13. 10.2217/fon-2018-0713 31858825

[11] Mangiameli G Crucitti P Rocco G. Microsized lung adenocarcinoma vs. small-sized lung adenocarcinoma: clinical characteristics, advantages and surgical implications. J Thorac Dis. (2016) 8(9):E1003–5. 10.21037/jtd.2016.08.04 27747046 PMC5059322

[12] Rho J Lee JW Quan YH Choi BH Shin BK Han KN Fluorescent and iodized emulsion for preoperative localization of pulmonary nodules. Ann Surg. (2021) 273(5):989–96. 10.1097/SLA.0000000000003300 30973387

[13] Kleedehn M Kim DH Lee FT Lubner MG Robbins JB Ziemlewicz TJ Preoperative pulmonary nodule localization: a comparison of methylene blue and Hookwire techniques. AJR Am J Roentgenol. (2016) 207(6):1334–9. 10.2214/AJR.16.16272 27657546

[14] Asamura H Kondo H Naruke T Tsuchiya R Wakao F Kaneko M Computed tomography-guided coil injection and thoracoscopic pulmonary resection under roentgenographic fluoroscopy. Ann Thorac Surg. (1994) 58(5):1542–4. 10.1016/0003-4975(94)91957-7 7979696

[15] Lizza N Eucher P Haxhe JP De Wispelaere JF Johnson PM Delaunois L. Thoracoscopic resection of pulmonary nodules after computed tomographic-guided coil labeling. Ann Thorac Surg. (2001) 71(3):986–8. 10.1016/S0003-4975(00)02505-4 11269486

